# DNA immunization as a technology platform for monoclonal antibody induction

**DOI:** 10.1038/emi.2016.27

**Published:** 2016-04-06

**Authors:** Shuying Liu, Shixia Wang, Shan Lu

**Affiliations:** 1 SL Consulting, Thousand Oaks, CA 91320, USA; 2 Department of Medicine, University of Massachusetts Medical School, Worcester, MA 01655, USA

**Keywords:** antigen delivery, DNA immunization, hybridoma, immunogenicity, monoclonal antibody

## Abstract

To combat the threat of many emerging infectious diseases, DNA immunization offers a unique and powerful approach to the production of high-quality monoclonal antibodies (mAbs) against various pathogens. Compared with traditional protein-based immunization approaches, DNA immunization is efficient for testing novel immunogen designs, does not require the production or purification of proteins from a pathogen or the use of recombinant protein technology and is effective at generating mAbs against conformation-sensitive targets. Although significant progress in the use of DNA immunization to generate mAbs has been made over the last two decades, the literature does not contain an updated summary of this experience. The current review provides a comprehensive analysis of the literature, including our own work, describing the use of DNA immunization to produce highly functional mAbs, in particular, those against emerging infectious diseases. Critical factors such as immunogen design, delivery approach, immunization schedule, use of immune modulators and the role of final boost immunization are discussed in detail.

## INTRODUCTION

The recent successful use of protective monoclonal antibodies as a life-saving treatment in Ebola virus-infected humans^1^ highlights the need for the development of new technologies that are fast and efficient in eliciting functional monoclonal antibodies (mAbs) to control emerging infectious diseases.

DNA immunization as it exists today was pioneered in the early 1990s. Its initial use as a vaccination platform generated great excitement due to the overall simplicity of using DNA plasmids to deliver immunogens.^2, 3, 4, 5^ One particularly attractive feature of DNA vaccines is that immunogens are produced *in vivo*, giving them the ability to induce T-cell immune responses through endogenous antigen processing and presentation pathways. However, the application of DNA immunization for human vaccine development has encountered challenges, specifically the low immunogenicity identified in early clinical studies, in which DNA vaccines were delivered via traditional needle injection without the use of adjuvants or other types of delivery instruments.

In recent years, significant progress has been made in the application of DNA vaccines in humans via two strategies: (i) the use of physical delivery methods, such as a gene gun or electroporation, which have greatly improved the immunogenicity of DNA vaccines in human volunteers,^6, 7^ and (ii) the development of a heterologous prime-boost scheme,^8^ in which the hosts are first immunized with a DNA vaccine, followed by boost immunizations with either recombinant protein antigens or traditional killed or live attenuated vaccines,^9, 10, 11^ which are more effective than homologous prime-boost immunizations using multiple doses of the same recombinant proteins or traditional vaccines.^12, 13, 14, 15, 16^

At the same time, it is generally agreed that DNA immunization is effective in small animal models, which can be useful for the production of high-quality mAbs. However, early animal model studies have focused mainly on T-cell immune responses, given the unique advantage of DNA immunization to induce such a response.^17, 18^ Little attention has been paid to the value of DNA immunization to elicit high-quality antibody responses, and much less has been given to the potential of DNA immunization to induce high-quality B-cell responses, which can be useful for the production of functional mAbs.

The current review provides a comprehensive summary of the literature that has accumulated over the past two decades, including our own work, in using DNA immunization to produce highly functional mAbs, in particular, against emerging infectious diseases. DNA immunization is more useful than traditional approaches to generating mAbs against more difficult targets, especially membrane proteins.

## DESIGN OF DNA VACCINES FOR MONOCLONAL ANTIBODY INDUCTION

DNA vaccines are constructed as mammalian expression vectors. Both the choice of expression vector and the design of immunogen inserts are important for the final antibody response, including the production of mAbs against the desired antigenic structures, but their roles are different in the process of DNA immunization.

### Optimal design of immunogen inserts

The *in vivo* immunogen expression feature of DNA vaccines offers a number of benefits.

Traditional protein-based immunization approaches have difficulty producing full-length protein immunogens by the recombinant protein method if the proteins are naturally expressed in a membrane-associated format, such as the multi-transmembrane G-protein coupled receptors (GPCRs) and ion channels. The DNA immunization approach can circumvent these problems because full-length proteins can be expressed *in vivo* when they are delivered in the form of DNA vaccines. Furthermore, it is well known that the structural integrity of proteins is critical for the induction of functional mAbs, yet these sensitive structures tend to be lost during the *in vitro* protein production process, regardless of whether they are produced as recombinant proteins or are extracted directly from cells or other sources in which the proteins are naturally expressed. Production of functionally active mAbs is highly dependent on the conformation of the proteins. Expressing intact immunogens *in vivo* by DNA immunization appears to have the best chance of inducing mAbs with the desired biological activities.

DNA vaccines possess the unique advantage of immunogen design flexibility. Immunogen inserts expressing the full-length sequences of target proteins are commonly used for all types of proteins, especially transmembrane proteins, with good success.^19, 20, 21, 22, 23, 24, 25, 26, 27, 28, 29, 30^ For intracellular proteins, one may assume that it is necessary to re-direct intracellular proteins into secretory pathways by adding a signal peptide to elicit a better antibody response. However, in a number of mAb production studies, native proteins have been used as DNA vaccine immunogen inserts without any sequence modifications.^25, 28, 31, 32^ On the other hand, the gene sequences of immunogen inserts for DNA vaccines can be easily edited to express ‘designer proteins.' For a single-transmembrane protein, the extracellular domain of a secretory protein can be selectively cloned as the immunogen insert when the objective is to generate mAbs against epitopes on the extracellular domain.^33, 34, 35, 36, 37^ Such an approach has also been applied to bacterial toxins. For example, a truncated fragment can be used for immunization in place of a full-length potentially lethal toxin protein, thereby avoiding the introduction of unwanted biological activity during the production of DNA vaccines, as well as during animal immunization.^38, 39^ Additional immunogen manipulations include the production of a ‘mini-gene insert' to express a short peptide sequence to cover a receptor-binding domain.^40^ In this case, antigenic determinants in the angiotensin-converting enzyme 2 binding domain of the severe acute respiratory syndrome spike protein, which does not closely match other coronaviruses, were predicted using software PROTEAN to induce anti-spike protein antibodies. Alternatively, a transmembrane anchor sequence can be added to non-membrane-associated antigens.^41^ As a simple and flexible immunogen design approach, DNA immunization offers a wide range of options to produce novel immunogen inserts for the induction of mAbs against even the most challenging targets (Table 1).

One unique feature of DNA immunization is the convenience of using the same DNA vaccine constructs to express antigens for mAb screening. The choice of reagents and methods depends on the original type of protein (Table 2). Cell-associated antigen-based screening has been widely and successfully used for mAbs targeting transmembrane proteins, viral envelope proteins and intracellular proteins. In these cases, cells expressing the immunogens were used without the need for protein purification to screen the binding activity of mAbs by either fluorescence-activated cell sorting analysis,^19, 20, 21, 23, 24, 26, 27, 29, 30, 34, 35^ whole cell enzyme-linked immunosorbent assay (ELISA),^33, 43, 48, 49^ or immunohistochemistry (IHC) methods.^25, 31, 32^ In one study, a novel in-cell Western screening method was developed and optimized to generate monoclonal antibodies against nuclear, cytoplasmic and transmembrane proteins.^28^

### Role of expression vectors

After two decades of effort by many research groups, the design of commonly used DNA vaccine vectors has been significantly optimized. It has been established that the promoter region of these DNA vaccine vectors is their most critical element and that the function of promoters can be further enhanced by other associated regulatory components. For example, the intron A sequence associated with the cytomegalovirus (CMV) promoter can significantly enhance the function of CMV promoter.^59^ The selection of poly-A tail is also important, as is optimized codon usage, which is particularly important for DNA vaccines expressing genes from infectious pathogens that often employ different codon usage than mammalian proteins.

One area that is less studied is whether sustained immunogen expression *in vivo* has a major impact on the induction of high-quality mAbs. In one study of antibody generation via intravenous delivery of plasmid DNA,^25^ the relative efficacy of CMV promoter and the human ubiquitin C promoter was compared using luciferase as the model immunogen. Using the ubiquitin promoter, which can lead to sustained antigen expression in the liver, resulted in significant levels of antibody titers seven weeks after a single hydrodynamic tail vein (HTV) delivery. In contrast, a single HTV delivery of the CMV promoter, which results in only short-term antigen expression, produced very low antibody titers over the same time frame. Nevertheless, both promoters enabled high antibody titers when delivered repeatedly. Thus, the study authors concluded that the choice of expression vector may reduce the number of genetic immunizations while still promoting the induction of high-titer antigen-specific antibodies.

Similarly, immune responses were compared after employing DNA vaccine plasmids encoding multi-drug resistant protein 4 (MRP4), a 12-transmembrane transporter, but with different promoters: the CMV early enhancer/chicken beta actin promoter, which is a strong synthetic promoter frequently used to constitutively drive high levels of gene expression in mammalian expression vectors, and the classical CMV promoter for transient expression.^30^ The immunogenicity results showed that the chicken beta actin promoter induced a higher antigen-specific immune response with HTV delivery, even when the plasmid was injected less frequently, compared with the use of a CMV promoter-containing plasmid.

## DELIVERY APPROACH AND SCHEDULE

### Physical versus chemical delivery approaches

Since the early discovery of DNA immunization, a wide range of delivery approaches has been studied. These approaches can be divided into two main categories. One is traditional needle injection of DNA plasmids in various solutions. Additional facilitating agents such as lipids and nanoparticles can be included in the solution to enhance delivery efficacy, and the composition of the chemical solution determines the uptake efficacy of the DNA vaccine plasmid. The second delivery type is based on physical forces. The most representative approach is the use of a gene gun, which uses a ‘ballistic' force to deliver the DNA plasmids. First, the DNA plasmids are coupled with gold particles, which are then delivered by the ballistic force to penetrate the cells of the targeted tissues. Early generation gene guns created ballistic forces by adding high-voltage electricity to a drop of water.^60^ More recent generation gene guns are based on the release of compressed gas.^61, 62^ Another physical method of DNA delivery is the use of electroporation. In this approach, DNA vaccines are first delivered by needle injection, followed by the application of an electrical current at the DNA injection site.

The relative immunogenicity of chemical and physical delivery approaches has been well analyzed. In one study, it was shown that electroporation delivery following intradermal needle injection was effective in delivering DNA vaccines to multiple intracellular compartments (that is, transmembrane, cytoplasm and nucleus), leading to the successful induction of mAbs.^28^ Another study showed that electroporation followed by intramuscular needle injection generated higher antibody responses than intramuscular needle injection alone. This approach was also more effective than intramuscular needle injection alone when the DNA vaccine was formulated with a chemical polymer and protein immunogen.^63^ In a more complete analysis comparing the delivery of DNA vaccines by intramuscular needle injection, electroporation following intramuscular needle injection, and gene gun alone, it was shown that both gene gun and electroporation delivery were more effective than the traditional intramuscular needle injection at eliciting higher antibody response levels.^64^ Both the gene gun and electroporation approaches are effective, but the gene gun approach requires only a few micrograms of DNA plasmid to achieve the same level of immune response elicited by the electroporation approach, which requires at least 100 μg of DNA plasmid at the first step of intramuscular injection, even in mice.

One interesting finding from literature is that although the delivery approach may be critical for the induction of high-level immune responses for human vaccine development, different DNA delivery approaches have been similarly successful in producing mAbs against a wide range of target antigens. Table 3 lists the mAbs elicited by the gene gun approach;^22, 24, 31, 32, 33, 34, 37, 43, 55, 57, 58^ needle injection, including intramuscular^19, 20, 23, 38, 41, 44,45,46, 48, 49, 50^ or intradermal^21, 35, 42^ injection; and electroporation following intramuscular or intradermal injection.^27, 28, 39, 52, 53, 56^

One unique but less-studied approach is hydrodynamic intravenous delivery. One large study compared the relative immunogenicity of HTV and hydrodynamic limb vein delivery methods with 18 different antigens, including different types of target antigens (that is, intracellular, transmembrane, and secretory). Both methods were successful, but the hydrodynamic limb vein delivery method was especially potent for generating antibodies against a wide range of targets.^25^ However, the hydrodynamic intravenous method may be more suitable for larger animals, such as rats and rabbits, given the larger vein size in these hosts compared with that in mice. The HTV method was also used successfully to generate a mAb against a 12-transmembrane transporter, which is a very challenging target for mAb induction.^30^

There have also been reports of producing mAbs with a single intrasplenic injection.^40, 54^ The author generated hybridomas by fusing spleen cells at 2, 3, 5, 10 and 25 days after a single intrasplenic injection of DNA vaccine plasmids. The highest number of specific hybridomas was generated at day 5 after a single initial injection.^54^ However, these mAbs appeared to be useful only for immunoblotting, and no additional studies were conducted to characterize their affinities.

### Immunization schedule

The optimal delivery schedule for the induction of high-quality mAbs by DNA immunization remains to be determined. The classical vaccine literature would indicate that an extended time period with long rest intervals may be more effective in eliciting high-quality antibody responses than immunizations in quick succession. There have been relatively few studies focusing solely on an optimal delivery schedule rather than the delivery approach itself. For example, it is not clear whether physical delivery approaches (that is, gene gun or electroporation) can be used more frequently than chemical delivery approaches (such as needle injection) because the former is more effective than the latter.

In most animal studies, the generation of mAbs requires multiple immunizations, usually every 2–3 weeks. However, a faster immunization procedure has been reported, which delivered 3–5 immunizations within 10–11 days at multiple sites by gene gun.^33, 57, 58^ In these studies, lymph nodes were used for fusion 48 h after the last injection without the need for a final boost. The mAbs generated were used for fluorescence-activated cell sorting, western blot analysis and enzyme-linked immunosorbent assay.

More studies are needed to further optimize the DNA immunization schedule to elicit high-quality mAbs. It is important to determine whether different DNA delivery approaches are optimal with certain delivery schedules and whether the same delivery schedule can be applied to different animal species.

## IMMUNE MODULATION

### Molecular adjuvants

Due to the low immunogenicity of DNA vaccines in early human studies, great effort has been devoted to the inclusion of various adjuvants as part of DNA vaccine formulation or immunization. Such studies have been widely reported in the literature, and the following section will review the use of molecular adjuvants only in the context of mAb induction. Adjuvants identified for previous vaccine development are likely to be useful for the induction of mAbs, but additional dedicated studies are needed to confirm the actual incremental benefits, particularly if DNA vaccine delivery is optimized, as discussed above.

*Escherichia coli* chaperone protein (GroEL) was demonstrated to act as a potent molecular adjuvant for DNA immunization and has been shown to enhance the Ab response against GPCRs, which are very difficult targets for mAb induction.^27, 65^ The authors reported that DNA immunization in mice with a plasmid encoding the full-length endothelin A receptor (ETAR) fused to GroEL at its C terminus induced strong, specific antibody responses to native ETAR. Co-injection of plasmids that expressed ETAR and GroEL (ETAR+GroEL) induced lower antibody responses than the ETAR–GroEL plasmid. In contrast, no specific antibody responses were produced in mice that were immunized with ETAR.^65^ The authors also mentioned that this strategy has been successfully applied to other GPCR targets and suggested that GroEL will be capable of producing antibodies against most GPCRs. One caution raised by the author is that in the case of unstable GPCRs, co-immunization with GroEL may be preferable to fusion with GroEL to induce an antibody response because fusion of some carrier proteins decreases their expression. Functional mAbs against a different GPCR target, the chemoCentryx chemokine receptor (CCX-CKR), were generated by the co-immunization of a plasmid encoding GroEL with a DNA vaccine plasmid-encoding target.^27^

In another study, a more complicated immunization strategy was tested, in which a plasmid encoding fetal liver tyrosine kinase 3 ligand was delivered as a priming dose, followed by the co-delivery of a plasmid encoding granulocyte-macrophage colony-stimulating factor and another plasmid encoding immunogen MRP4. This study demonstrated that the addition of fetal liver tyrosine kinase 3 ligand and granulocyte-macrophage colony-stimulating factor as immune modulators significantly improved not only the overall immune response in the mice but also the induction of antibodies capable of recognizing native extra-cellular epitopes.^30^

### DNA prime-protein boost

One approach that presents a great advantage for the induction of high-titer and high-quality antibody responses is the heterologous prime-boost approach. In this approach, the DNA vaccine is delivered as the priming immunization, followed by a boost with protein antigens as recombinant proteins, peptides, or traditional inactivated or live attenuated vaccines. One unexpected finding regarding DNA priming immunization is their ability to induce higher-level antigen-specific B-cell responses.^66^ Our research group has shown that DNA primer immunization was more effective than protein immunization in activating germinal center B cells. Higher levels of antigen-specific B cells set the stage for more robust antibody responses. Whether this higher-level activation of B cells leads to better mAb cloning remains to be determined.

The DNA prime-protein boost approach has been used to generate mAbs in both mouse and rabbit models. This approach was effective in generating a panel of mAbs that are protective against *Clostridium difficile* toxin A challenge, as well as mAbs for use as sensitive reagents to detect toxin A in various testing samples.^39^ We also reported the use of DNA immunization to generate rabbit mAbs with a high affinity for and a diverse epitope-binding profile to the human immunodeficiency virus type 1 (HIV-1) envelope antigen.^37^ One unique rabbit mAb targeted an area on the envelope protein of HIV-1, which blocks the binding of CD4 and the HIV-1 receptor.^67^ Another research group used a similar DNA prime-protein boost approach to generate a higher antibody titer and higher quality mAbs than those observed with protein immunization alone.^36^

## ROLE OF FINAL BOOST IMMUNIZATION

Production of mAbs by the traditional hybridoma method requires the availability of many activated antigen-specific B cells in lymphoid organs for fusion. With the traditional protein immunization approach, this is achieved by a final intravenous or intraperitoneal injection 3–5 days before fusion. This same procedure may also be needed for DNA immunization.

Hybridoma fusions using cells from DNA-immunized animal hosts without a final boost have been reported by several groups.^38, 41, 44, 46, 55^ However, the overall fusion efficiency was low, and the resulting antibodies had low binding affinity, with IgM as the dominant isotype. One study compared the ability of a final protein boost with no final protein boost with respect to hybridoma generation and concluded that despite significant antibody responses in the immunized animals, the fusion of mouse spleen cells yielded a low number of and low-quality hybridomas unless the mice were given a boost 3–5 days before fusion.^21^

Other studies included an additional DNA plasmid immunization by intramuscular or intradermal injection 3–5 days before fusion as a final boost.^42, 45, 51^ Although the numbers of mAbs generated were small (that is, only a few mAbs from each fusion), mAbs with good binding affinity and diversity were reported.^45, 51^ The final DNA plasmid boost could also be delivered by hydrodynamic injection five days before fusion, and specific mAbs were successfully generated,^25^ including some against very difficult targets, such as multi-transmembrane proteins.^27, 30^

Proteins are commonly used as final boost reagents. Purified protein has been used successfully for the final protein boost for both secretory proteins^47, 56^ and intracellular proteins.^54, 57, 58^ For single-transmembrane proteins or glycophosphatidylinositol-anchored proteins, a purified extracellular domain can be used for the final boost if it is confirmed that the proteins retain a native conformation.^34, 37, 43^ Furthermore, cells expressing antigen proteins have been used directly as the final boost. This approach was successful for both membrane proteins^19, 20, 21, 23, 24, 35, 48, 49^ and intracellular proteins.^25, 28, 31, 32^ The use of cells as a boosting reagent can work for secretory proteins that are difficult to purify by adding a glycophosphatidylinositol anchor. In the case of viral antigens, inactive viral particles have been used successfully as the final boost.^51,52,53^ Table 4 provides a summary of final boost immunization options.

DNA immunization can be used to generate mAbs by either a traditional hybridoma approach or the single B-cell cloning approach in different animal models. Our research group has recently produced mAbs from human volunteers who were immunized via an HIV vaccine DNA prime-protein boost regimen using a single B-cell cloning method (paper in preparation). These results show that DNA immunization can be used in a wide range of hosts to produce high-quality mAbs.

## SUMMARY

DNA immunization is a powerful approach to producing high-quality mAbs and offers several unique advantages: (i) DNA immunization is an efficient method of testing different immunogen designs; (ii) DNA immunization does not require the production or purification of proteins from a pathogen, which avoids any issues related to biosafety; (iii) DNA immunization allows a rapid response to an emerging infectious agent once the pathogen gene sequence is known; (iv) DNA immunization is effective in generating mAbs against conformation-sensitive targets; (v) DNA immunization can be used for mAb induction in a wide range of hosts, including mouse, rabbit and human.

## Figures and Tables

**Table 1:** Types of DNA vaccine immunogens used for mAb induction

**Immunogen inserts**	**Original types of proteins**	**References**
Full length	Transmembrane	^19, 20, 21, 22, 23, 24, 25, 26, 27, 28, 29, 30^
	Extracellular matrix	^42^
	GPI anchor	^43^
	Intracellular	^25, 28, 31, 32^
	Secretory	^25, 44, 45, 46, 47^
	Viral	^25, 48,49, 50, 51, 52, 53^
	Bacteria	^54^
Extracellular domain	Single transmembrane	^33, 34, 35, 36, 37^
Fragment/subunit	Any type	^25, 38, 39, 55^
Mini-gene insert	Any type	^40^
Novel format, vaccibody	Secretory	^56^
Immunogen-transmembrane domain fusion	Viral non-structure	^41^
Immunogen-Fc fusion	Intracellular	^57, 58^

Abbreviation: glycophosphatidylinositol, GPI.

**Table 2:** Screening methods

**Screening reagent**	**Original type of protein**	**Screening method**	**References**
Proteins	Secretory	ELISA	^37, 38, 39, 40, 42, 44, 45, 46, 47, 50, 51, 54, 55, 56^
	Intracellular	ELISA	^36, 57, 58^
	Single transmembrane	ELISA	^21, 22^
Cells expressing immunogens	Transmembrane	FACS	^19, 20, 21, 23, 24, 26, 27, 29, 30, 34, 35^
		Whole cell ELISA	^33, 43^
	Viral envelope	Whole cell ELISA	^48, 49^
		Immunostaining	^53^
	Intracellular	In-cell western	^28^
		IHC	^25, 31, 32^
Viral particles	Viral	Dot enzyme immunoassay	^41^
		ELISA	^52^

Abbreviation: enzyme-linked immunosorbent assay, ELISA; fluorescence-activated cell sorting, FACS; immunohistochemistry, IHC.

**Table 3:** DNA vaccine delivery approaches used for mAb induction

**Delivery approaches**	**Original types of protein**	**References**
Gene gun	Single transmembrane (Flt-3R)	^33^
	Intracellular (PED/PEA-15)	^57^
	Intracellular (annexin-V)	^58^
	Single transmembrane (CAR)	^22^
	Two- transmembrane (P2X7)	^24^
	GPI anchored enzyme	^43^
	Intracelluar (BCL-6)	^31^
	Intracelluar (MALT1)	^32^
	Single transmembrane (MHCI-related gene A)	^34^
	Parasite lipoprotein	^55^
	Viral envelop (HIV gp120)	^37^
IM	Bacteria toxin (Helicobacter pylori vacuolating cyto toxin)	^38^
	Seven transmembrane, GPCR (TSHR)	^19^
	Viral envelop (HGV E2)	^48^
	Seven transmembrane, GPCR (TSHR)	^20^
	Viral non-structure (Dengue NS1)	^41^
	Viral envelop (H5N1)	^49^
	Secretory protein, enzyme (prostate-specific antigen)	^45^
	Viral surface (HBV preS2/S)	^50^
	Seven transmembrane, GPCR (TSHR)	^23^
	Secretory protein, cytokine (CKLF1)	^44^
	Secretory protein, cytokine (Interferon beta)	^46^
ID	Single transmembrane (RET and CD30)	^21^
	Extracellular matrix and plasma glycoprotein (Fibulin-1)	^42^
	Single transmembrane (CD2)	^35^
IM followed by EP	Seven transmembrane, GPCR (CCX-CKR)	^27^
	Viral envelop (H5N1)	^52^
	Secretory (mCherry)	^56^
	Viral envelop (H1)	^53^
	Bacteria toxin (*C. difficile* toxin A)	^39^
ID followed by EP	Multiple targets (transmembrane, intracellular)	^28^

Abbreviations: B-cell lymphoma 6 protein, BCL-6; coxsackievirus and adenovirus receptor, CAR; cluster of differentiation 2, CD2; cluster of differentiation 30, CD30; chemokine-like factor 1, CKLF1; electroporation, EP; fetal liver tyrosine kinase 3 receptor, Flt-3R; hepatitis B virus, HBV; hepatitis G virus E2 protein, HGV E2; intradermal, ID; intramuscular, IM; mucosa-associated lymphoid tissue lymphoma translocation gene 1, MALT1; P2X purinoceptor 7, P2X7; phosphoprotein over expressed in diabetes/phosphoprotein enriched in astrocytes, PED/PEA-15; rearranged during transfection, RET; thyroid stimulating hormone receptor, TSHR.

**Table 4:** Options for the final boost

**Type of boost**	**Original types of proteins**	**References**
DNA by IM or ID	Any type	^42, 45, 51^
DNA by HTV	Any type	^25, 27, 30^
Protein	Secretory	^39, 47, 56^
	Intracellular	^54, 57, 58^
	Single-transmembrane/GPI anchored	^34, 37, 43^
Cells expressing immunogen	Transmembrane	^19, 20, 21, 23, 24, 35, 48, 49^
	Intracellular	^25, 28, 31, 32^
Viral particle	Viral surface	^51–53^

Abbreviations: hydrodynamic tail vein, HTV; intradermal, ID; intramuscular, IM.
